# “King of the forage”—Alfalfa supplementation improves growth, reproductive performance, health condition and meat quality of pigs

**DOI:** 10.3389/fvets.2022.1025942

**Published:** 2022-11-02

**Authors:** Jixiang Ma, Weikang Huangfu, Xu Yang, Junying Xu, Yan Zhang, Zhichang Wang, Xiaoyan Zhu, Chengzhang Wang, Yinghua Shi, Yalei Cui

**Affiliations:** ^1^College of Animal Science and Technology, Henan Agricultural University, Zhengzhou, China; ^2^Henan Key Laboratory of Innovation and Utilization of Grassland Resources, Zhengzhou, China; ^3^Henan Forage Engineering Technology Research Center, Zhengzhou, China

**Keywords:** alfalfa, pig, dietary fiber, production performance, intestinal health

## Abstract

As one kind of high-quality feed with rich nutrients, including high quality protein and amino acids, dietary fiber, enriched vitamins and mineral elements and bioactive molecules, alfalfa has been widely used in the production of ruminant livestock. As the understanding of alfalfa becomes more and more comprehensive, it is found that the high-quality nutrients in alfalfa could have positive effects on pigs. An increasing number of researches have shown that supplementing dietary alfalfa to the diet of gestating sows reduced constipation, alleviated abnormal behavior, improved satiety and reproductive performance; supplementing dietary alfalfa to the diet of piglets improved growth performance and intestinal barrier function, reduced intestinal inflammatory response and diarrhea; supplementing dietary alfalfa to the diet of growing-fattening pigs improved production performance and pork quality. Moreover, the mechanisms by which various nutrients of alfalfa exert their beneficial effects on pigs mainly including dietary fiber stimulating intestinal peristalsis, enhancing the activity of digestive enzymes, and promoting the colonization of beneficial bacteria in the intestinal tract through fermentation in the intestine, producing short-chain fatty acids and thus improving intestinal health; high quality protein and amino acids are beneficial to improve animal health condition; rich vitamins and mineral elements play an important role in various physiological functions and growth and development of the body; and bioactive molecules can improve the antioxidant and anti-inflammatory level. Therefore, alfalfa could be used as pig feed ingredient to alleviate various problems in the pig industry and to improve pig production performance. In this review, we detail the current application of alfalfa in pigs and discuss the potential mechanisms involved in how alfalfa improves growth and reproductive performance, pork quality, and intestinal health of the animals, thus laying the foundation for the increased application of high-quality forage in pig production.

## Introduction

The current intensive farming model of animal husbandry has brought about many negative problems, such as the decline in reproductive performance of sows (1), the increase of diarrhea in piglets (2) and the deterioration in pork quality (3). Especially after the implementation of the “antibiotic ban”, a series of problems have emerged in the pig breeding industry. The development of grain-saving healthy breeding has become an important goal in the current animal husbandry.

Alfalfa (*Medicago sativa* L.) is one kind of perennial herb belonging to the legume family (4) that is rich in nutrients, including protein, dietary fiber, vitamins, and minerals (5). In addition, alfalfa contains a variety of bioactive molecules and unknown growth factors, including saponins, polysaccharides, flavonoids and other bioactive molecules. Consequently, alfalfa is known as the “king of forage grass”. In the past, it has been found that supplementing alfalfa hay to cow diets increased milk yield (6), and that increasing the amount of alfalfa hay supplementation in the cow's diet shortened the interval to conception (7). In recent years, more and more studies have found that adding alfalfa to pig diets at different stages can help to improve pig performance. In this review, we summarize the nutritional value of alfalfa, including the positive physiological effects of its dietary fiber, and detail the current application of alfalfa in pigs. We further discuss the potential mechanisms underlying how alfalfa improves animal growth and reproductive performance, intestinal health, and pork quality, thus laying the foundation for the increased application of forage in pig production.

## The nutritional value of alfalfa

### Alfalfa is rich in crude protein, vitamins, minerals, and other bioactive molecules

Alfalfa is rich in essential nutrients such as protein, vitamins, minerals, and fiber, in addition to as yet unidentified growth factors required by animals. The level of crude protein is one of the most important indicators of the nutritional value of forage. The level of crude protein in alfalfa is relatively high, generally about 18–20% (8). The lysine, tryptophan and other amino acids in alfalfa have a reasonable pattern and relatively balanced composition ratio, which are easily digested and utilized by animals. Alfalfa is also rich in lysine and methionine, about 0.80 and 0.23% (9), respectively, which is beneficial to balance the deficiency of lysine in grain feed. Alfalfa has an abundance of beneficial fatty acids, of which the amount of α-linolenic acid and linoleic acid are higher and oleic acid is lower (10). α-Linolenic acid can help lower the risk for cardiovascular disease and the occurrence of hyperlipidemia (11). Moreover, linoleic acid plays an important role in maintaining the permeable barrier of epidermis, and it is a precursor component for the synthesis of various bioactive molecules such as prostaglandins and leukotrienes (12). Oleic acid can cause neutrophils to produce reactive oxygen species (ROS) and induce leukocyte death (13). Besides, alfalfa is also rich in vitamins, including folic acid; vitamins B, C, E, and K; and carotene (14, 15). Furthermore, the calcium, magnesium and potassium content of alfalfa meal concentrate is ~21, 7.9, and 23 mg/g, respectively (16). High levels of calcium and potassium have bone-strengthening effects, while magnesium can improve immune function (17). Alfalfa also contains bioactive molecules, including polysaccharides, saponins, and flavonoids, in addition to as yet unidentified growth factors. Alfalfa polysaccharides possess a variety of properties, including exerting immunomodulatory and anti-inflammatory effects and promoting the proliferative transformation of lymphocytes (18). Alfalfa saponins have been shown to stimulate cholesterol excretion and also exhibit anti-inflammatory, antioxidant, and antibacterial properties (19). Meanwhile, numerous studies have found that flavonoids have physiological effects such as anti-cancer, promoting animal growth and enhancing the immunity (20). Alfalfa is also rich in unidentified growth factors that may be beneficial for animal production performance.

### Alfalfa is a source of high-quality dietary fiber

The term “dietary fiber” was first introduced by Hipsley in 1953 (21). It is now generally accepted that dietary fiber refers to polysaccharides primarily containing resistant starch, oligosaccharides, fructans, pectins, cellulose, hemicellulose, and lignin, which cannot be directly absorbed and utilized by animals. Southgate (1978) classified fiber into two types using chemical classification: structural fiber including non-fibrous polysaccharides, cellulose and lignin and nonstructural fiber including pectins, gums, algal polysaccharides and denatured fiber (22). Depending on the solubility of the fiber, dietary fiber can be divided into insoluble dietary fiber (IDF) and soluble dietary fiber (SDF) (23).

The average crude fiber content in alfalfa hay is ~24.8% (24), with insoluble dietary fiber, such as cellulose, hemicellulose, xylan, and lignin, accounting for more than 90% of the total (25, 26). Regarding chemical classification, in alfalfa hay, non-structural carbohydrates such as sugar, pectin, and starch comprise 20–35% and structural carbohydrates such as cellulose, hemicellulose, and lignin 30–50% of the total crude fiber content (27). Most of the dietary fiber in alfalfa can be degraded and absorbed by the intestinal flora of animals, whereas lignin, which is difficult to degrade and utilize, accounts for only a relatively small proportion (28–30). These observations indicate that alfalfa can serve as a high-quality fiber source feed.

### The digestion and utilization of dietary fiber by pigs

In the past, dietary fiber was widely used in ruminant production due to the commonly held belief that the gastrointestinal tract of monogastric animals was weak in fiber decomposition, and it was once thought to reduce the digestibility of feed for monogastric animals and reduce their production performance. In recent years, with the increasing awareness of dietary fiber, it has been referred to as the “seventh macronutrient”. With the continuous deepening of research on fiber nutrition, dietary fiber has been found to have certain physiological nutritional functions for pigs (31). The IDF can stimulate peristalsis, reduce intestinal stagnation and increase digestive enzyme activity in pigs, thus increasing gastrointestinal feed intake, which in turn increases the passage rate of digestive liquids in the digestive tract to reduce intestinal infections and pathogens adhering to the mucosa of the gastrointestinal tract (32). The SDF is easily fermented and decomposed by the microbiota in the animal's gastrointestinal tract, improving the intestinal flora, promoting the colonization of beneficial bacteria in the intestinal tract and the production of short-chain fatty acids (SCFAs), while reducing the production of harmful bacteria and harmful molecules.

As monogastric omnivores, pigs have markedly different gastrointestinal tract structure and digestive physiology from that of ruminants such as cattle and sheep, and thus differ greatly in roughage usage. Digestion and absorption of nutrients in pigs occurs mainly in the gastrointestinal tract (33). Once feed enters the digestive tract, it must undergo physical, chemical, and microbial digestion, which transform it into structurally simple soluble molecules such as amino acids, glycerol, fatty acids, and glucose so that it can be absorbed and utilized. The digestive tract of pigs is rich in microbiota, most of which is present in the large intestine (34). The fermentation of dietary fiber by pigs occurs mainly in the cecum and colon, and the fibrinolytic bacteria that exist in the porcine colon mainly comprise members of the *Lactobacillus* and *Bifidobacterium* genera (35). The number of fibrinolytic bacteria increases as the pig grows (36). Indeed, adult pigs having ~6.7-fold the number of bacteria as growing pigs (37) and, accordingly, have a greater capacity for digesting dietary fiber. The fiber content of feed also has a significant influence on the composition of the intestinal microbiota, and feed with high levels of fiber can increase the number and activity of fiber-degrading bacteria in the large intestine of sows (38). Combined, these findings suggest that the high dietary fiber content of alfalfa can greatly enhance animal performance.

## The application of alfalfa in pig production

### The application of alfalfa in sows

#### Alfalfa can reduce sow constipation

Constipation refers to the absence of normal defecation within a certain period of time, manifesting as difficulty in defecating, abdominal pain, and abdominal distension (39). Pregnant sows are very prone to constipation because gastrointestinal motility is weakened during pregnancy and transit time is significantly prolonged, resulting in increased water absorption and ultimately sow constipation (40). Constipation can lead to abdominal distention, intestinal obstruction and prolonged farrowing, as well as influence the number of stillborn, which can affect the health and performance of the sow (41, 42). The addition of fiber to the sow diet can promote gastrointestinal motility, increase the water-holding capacity of the feces, and reduce the digestive transit time of the feces in the intestine (Table 1). Baker et al. found that adding 5% dehydrated alfalfa meal to the diet of pregnant gilts increased the softness of sow feces (43), thereby relieving constipation. Krogh et al. also found that supplementing an appropriate amount of alfalfa meal to the diet of gestation sows improved the softness of the feces (44). The reduction of constipation in pregnant sows can improve the overall performance of the sows (45), which improves fetus delivery by the sows as well as the survival rate of piglets (42).

**Table 1:** The effects of dietary alfalfa supplementation on health condition of gestation sow.

**Feeding stage**	**Alfalfa addition form**	**Amount**	**Main results**	**References**
Pregnant gilts	Dehydrated alfalfa meal	5%	The softness of pregnant sow feces↑	(43)
Gestation sows	Alfalfa meal	16.9%	The softness of the gestation sow feces↑	(44)
Gestation sows	Alfalfa-orchardgrass hay meal	46%	Backfat deposition during gestation ↓;Weight loss from 109th days of gestation to 14th days postpartum↑	(46)
Gestation sows	Dehydrated alfalfa meal	As the main feed material	The gestation sows daily weight gain↓	(47)
Gestation sows	Alfalfa meal	50 or 95%	Daily weight gain in gestation sows↓	(48)

#### Alfalfa can increase sow satiety and reduce abnormal behavior

Too much energy in the diet of a gestating sow can result in excess body weight and backfat thickness. This may lead to a variety of problems during delivery (71). To ensure optimal health and optimal physiological conditions for gestating sows, as well as to reduce farming costs, gestating sows are often fed restrictively; however, restrictive feeding can lead to abnormal stereotyped behaviors such as chewing without food, loud growling, climbing on bars, licking troughs and floors, and biting bars due to starvation (72). Feed with a high level of fiber has a lower energy content than other feed materials such as wheat and maize and supplementing fiber-rich feed materials to the diet of gestating sows makes it easier to control their daily energy intake (73). Fiber feed can increase satiety and reduce hunger-induced abnormal behavior in sows (74). Holzgraefe et al. found that the addition of 46% alfalfa-orchard grass hay meal to the diet of gestation sows significantly reduced backfat deposition during gestation and more weight loss from 109th days of gestation to 14th days postpartum (46). Libal et al. used dehydrated alfalfa as the main feed material for gestation sows and found a reduction in daily weight gain (47). Calvert et al. also found that adding either 50% or 95% alfalfa meal to gestation sow diets reduced daily weight gain in gestation sows (48). These results are summarized in Table 1. The supplementation of sow diets with an appropriate amount of alfalfa meal increased the abundance of fibrinolytic bacteria in the gut (75). Additionally, the fiber in alfalfa meal can be fermented by fibrinolytic bacteria in the living gut, thereby producing SCFAs that can provide ~30% of the maintenance energy (76). Thus, the addition of an appropriate level of alfalfa meal to the diet of gestating sows can enhance satiety, provide maintenance energy, and reduce abnormal behavior. This, in turn, has beneficial effects on the survival of newborn piglets.

#### Alfalfa can improve the reproductive performance of sows

The excessive consumption of dietary fiber during gestation may have adverse effects on sows owing to its anti-nutritional effects. However, in the right proportion, dietary fiber can improve reproductive performance (77, 78) and reduce the mortality of piglets before weaning (Table 2). Danielson and Noonan found that the addition of 96.75% alfalfa hay to the diet of gilts during their first three successive gestation periods significantly increased the number of piglets born live per litter, individual birth weight, and individual weaning weight in the third generation of sows, and each of the generations maintained a farrowing percentage of ~95% (49). Liu et al. found that the addition of a certain percentage of alfalfa meal to the diet of gestation sows significantly reduced intrauterine growth restriction (IUGR), increased lactation feed intake, reduced intestinal inflammatory factors in sows, raised the amount of intestinal anti-inflammatory flora and improved body function in sows, which could further enhance piglet condition through vertical transmission compared to the normal diet group (50). Teague found that supplementing 18% sun-cured alfalfa to sow diets before breeding and during gestation increased the number of corpora lutea in sows, significantly increased litter numbers and increased the number of piglets that survived weaning (51). Pollmann et al. found that supplementing 50% alfalfa to the diet of gestation sows significantly reduced weight gain during gestation and significantly increased the number of piglets alive at 14 days, and supplementing alfalfa to the diet improved the total survival rate of piglets at 14 days by ~8% over three reproductive cycles of sows (52). Seerley and Wahlstrom supplemented different percentages of dehydrated alfalfa meal to the diet of 200 lb sows until the end of lactation and found that the addition of 10% dehydrated alfalfa meal to the sow's diet increased the number of weaned piglets and had the highest average litter weight of piglets (53). Meanwhile, the addition of alfalfa–orchard hay and alfalfa hay to the diets of gestating sows increased the number of live piglets by between 0.1 and 0.8 (79). Combined, these data suggest that the supplementation of the right proportion of alfalfa to the diet of gestating sows can improve their reproductive performance.

**Table 2:** The effects of dietary alfalfa supplementation on the reproductive performance of gestation sow.

**Feeding stage**	**Alfalfa addition form**	**Amount**	**Main results**	**References**
First three successive gestation periods	Alfalfa hay	96.75%	The number of piglets born live per litter, individual birth weight, and individual weaning weight in the third generation of sows ↑	(49)
Gestation	Alfalfa meal	10%	IUGR ↓;Intestinal inflammatory factors ↓; Lactation feed intake ↑; Intestinal anti-inflammatory flora ↑	(50)
Before breeding and during gestation	Sun-cured alfalfa	18%	The number of corpora lutea ↑; Litter numbers ↑; The number of piglets that survived weaning ↑	(51)
Gestation	Alfalfa	50%	Weight gain ↓; Piglets alive at 14 days ↑; The total survival rate of piglets at 14 days over three reproductive cycles of sows ↑	(52)
Gestation until the end of lactation	Dehydrated alfalfa meal	10%	The number of weaned piglets ↑; Average litter weight of piglets ↑	(53)

### The application of alfalfa in piglets

#### Alfalfa can influence the growth performance of piglets

Alfalfa may be supplemented as a high-quality feed ingredient in piglet diets given its abundance of nutrients (Table 3). Adams et al. supplemented the diets of weaned piglets with different percentages of alfalfa and found that the 12% alfalfa group increased average daily gain (ADG) and average daily feed intake (ADFI) and significantly reduced piglet diarrhea compared to other groups, further improving piglet growth performance (54). Another study showed that the supplementation of alfalfa fiber in the diet of weaned piglets increased blood albumin, globulin, and total protein levels; reduced the cholesterol level; increased intestinal villus height and the ratio of villus height to crypt depth; and improved piglet performance (80). Additionally, Liu et al. demonstrated that the addition of 5% alfalfa meal to the diets of piglets significantly reduced the ratio of diarrhea, tended to reduce mortality, and had a beneficial effect on the intestinal flora (55). Meanwhile, another study reported that adding 5% alfalfa fiber to the diet of piglets with lipopolysaccharide-induced intestinal injury increased the ADG and the gain: feed (G:F) ratio and improved the growth of the piglets (56). However, too high a content of dietary fiber may also negatively influence the digestion of nutrients by the piglets. Freire et al. supplemented the diet of weaned piglets with 20% alfalfa meal and found that the digestive transit time was reduced, as were the digestibility of neutral detergent fiber (NDF) and acid detergent fiber (ADF) (57). Moore et al. also supplemented the diets of piglets with 20% alfalfa meal and found a reduction in the apparent digestibility of dry matter, nitrogen and energy (58). Stanley et al. added dehydrated alfalfa meal to piglets' basal diets and reduced the digestibility of dry matter, nitrogen and energy (59). Therefore, an appropriate amount of alfalfa should be supplemented to the piglets' diet, otherwise it would affect the piglets' digestion and absorption of nutrients, reducing growth performance.

**Table 3:** The effects of dietary alfalfa supplementation on the growth performance and diarrhea of piglet.

**Feeding stage**	**Alfalfa addition form**	**Amount**	**Main results**	**References**
Weaned piglets	Alfalfa	12%	ADG ↑; ADFI ↑; Piglets diarrhea ↓	(54)
Piglets	Alfalfa meal	5%	The ratio of diarrhea ↓; Piglet mortality ↓	(55)
Piglets	Alfalfa fiber	5%	ADG ↑; G:F ↑; SCFAs ↑; The relative abundance of cellobiolytic and anti-inflammatory bacteria ↑; Piglets diarrhea ↓	(56)
Weaned piglets	Alfalfa meal	20%	The digestive transit time ↓; The digestibility of NDF and ADF ↓	(57)
Piglets	Alfalfa meal	20%	The apparent digestibility of dry matter, nitrogen and energy ↓	(58)
Piglets	Dehydrated alfalfa meal	Fed at 0, 1 or 2% of body weight daily.	The digestibility of dry matter, nitrogen and energy ↓	(59)
Suckling piglets	Alfalfa	1.3%	The abundance of *Coprococcus eutactus* ↑; The abundance of potential pathogen *Streptococcus suis* ↓; Butyric acid in the intestine ↑	(60)

#### Alfalfa can reduce diarrhea of piglets

The digestive system of piglets is not well-developed. The gastrointestinal tract lacks the corresponding digestive enzymes, the intestinal flora is incomplete, and immune function and body temperature regulation are weak. As piglets are gradually weaned, various stresses, such as dietary stress, lead to intestinal dysfunction (81) and inflammation. The resulting diarrheal diseases and diminished piglet performance may lead to reduced feed intake and even death. Alfalfa is rich in saponins, polysaccharides, flavonoids and other active factors, which have the ability to resist harmful bacteria and eliminate inflammation, improving the immunity of piglets, reducing the rate of diarrhea and promoting animal growth (82). Zhang et al. added 1.3% alfalfa to the supplements of suckling piglets and found that the composition of the intestinal flora of the piglets was improved, as evidenced by the increased abundance of *Coprococcus eutactus*, the reduced abundance of the potential pathogen *Streptococcus suis*, the increased production of butyric acid in the intestine, and the reduced production of molecules harmful to the intestine, thereby reducing intestinal inflammation and protecting intestinal health (60). Sun et al. found that the addition of 5% alfalfa fiber to the diets of piglets with lipopolysaccharide-induced injury increased the relative abundance of cellobiolytic and anti-inflammatory bacteria in the intestines, increased the level of intestinal SCFAs, and inhibited the inflammatory response, thus improving the intestinal health and reducing the rate of diarrhea in the piglets (56). Overall, the supplemented appropriate amount of alfalfa in piglet diets may improve the intestinal flora of piglets (83), increasing the intestinal beneficial bacteria and fibrinolytic bacteria, increasing the content of intestinal SCFAs and enhancing the intestinal barrier function, thus reducing intestinal inflammation and diarrhea in piglets.

### The application of alfalfa in growing-fattening pigs

#### Alfalfa can improve the growth performance of growing-fattening pigs

The digestion and utilization of protein, amino acids, and other nutrients in alfalfa by pigs does not differ from that of other feed nutrients. Adding fiber feed to the pig diet is always controversial given that they are monogastric animals (84). Growing–fattening pigs have a relatively well developed hindgut with a large number of microorganisms that can degrade fiber in the colon. Accordingly, the fiber component of alfalfa can be degraded and absorbed by the animals, at least to a certain extent. Studies have shown that the provision of a high fiber diet can enhance the ability of pigs to digest fiber (Table 4). For instance, Škrlep et al. supplemented alfalfa hay in the barrows organic food diet, the final live weight and backfat thickness of pigs did not differ from the normal group, but there was a tendency to increase the ADG of pigs (61). Bohman et al. found growing-fattening pigs can increase weight gain of 1.3–1.7 pounds per day with 30–50% alfalfa supplementation (62). Chen et al. found that the pH in ileal digesta and the concentrations of acetate, propionate, and total volatile fatty acids in the feces increased with the addition of 5, 10, and 20% alfalfa meal to the diet of growing pigs (63). Wang et al. added 15% alfalfa meal to growing pigs' diets, which had a tendency to reduce ADIF and significantly increased the G:F ratio, significantly increased butyrate concentration in cecum and increased intestinal flora evenness (64). The microbiota in the intestinal tract of pigs can utilize the dietary fiber in alfalfa to increase the feed conversion rate and improve the production performance. Kozera et al. supplemented green alfalfa forage to the diet of growing-finishing pigs, which reduced the daily water intake and achieved a relatively satisfactory G:F ratio, as well as increased high-density lipoprotein (HDL) cholesterol level in the serum and the final body weight was also slightly increased (65).

**Table 4:** The effects of dietary alfalfa supplementation on the growth performance of growing-fattening pig.

**Feeding stage**	**Alfalfa addition form**	**Amount**	**Main results**	**References**
Barrows	Alfalfa hay	Free feeding	ADG ↑	(61)
Growing-fattening	Alfalfa	30% to 50%	The weight gain of 1.3 to 1.7 pounds per day	(62)
Growing	Alfalfa fiber	5%, 10% and 20%	The pH in ileal digesta and concentration of acetate, propionate, and total volatile fatty acid in the feces ↑	(63)
Growing	Alfalfa meal	15%	ADIF ↓;The G:F ratio ↑; Butyrate concentration in the cecum and intestinal flora evenness↑	(64)
Growing-finishing	Green alfalfa forage	3 kg per pen	The daily water intake ↓;HDL cholesterol level in the serum ↑	(65)

#### Alfalfa can improve pork quality

In addition to growth performance, the pork quality of fattening pig is also an important indicator in pig farming production (85). Fiber-rich feed sources can have a beneficial effect on pork quality in fattening pigs (Table 5). Stevenson et al. found that the addition of dehydrated alfalfa meal to the diets of pigs helped to improve the grade and length of carcasses (66). TomaŽin et al. found that supplementation of alfalfa hay in the barrows organic food diet significantly increased the pH value at 45 min after slaughter and significantly decreased the pH value at 24 h after slaughter, significantly increased the proportion of monounsaturated fatty acids and decreased the proportion of saturated fatty acids in intramuscular fat of longissimus lumborum muscle, and significantly increased the proportion of polyunsaturated fatty acids in the backfat, which improved the quality of the pork (67). Bohman et al. found that the addition of different percentages of alfalfa to fattening pig diets significantly reduced the percentage of weight gain, slaughter rate, depth of back fat in the carcass and significantly increased the percentage of shoulder, ham and loin in the carcass as the alfalfa content of the diets increased, and that pigs with 50% addition of alfalfa to their diets had a higher percentage of lean meat (68). Kidwell et al. also found that the addition of 50% alfalfa to growing-fattening pig diets resulted a higher proportion of lean meat in pork (69). Karwowska et al. added 0.2% alfalfa extract to the diets of fattening pigs, which did not lead to the deterioration of meat quality (86). Another study by the same authors reported that the addition of 0.2% alfalfa extract to the diets of fattening pigs accelerated growth and development and improved sensory evaluation scores for ham flavor and consistency (70). In conclusion, the addition of alfalfa to the diets of pigs can help increase the polyunsaturated fatty acid content in pork (87) and increase the proportion of lean meat, thus improving pork quality.

**Table 5:** The effects of dietary alfalfa supplementation on the pork meat quality.

**Feeding stage**	**Alfalfa addition form**	**Amount**	**Main results**	**References**
Weaned pigs reached weights of 100, 125 or 150 lb.	Dehydrated alfalfa meal	Different levels of Alfalfa	Grade and length of carcasses ↑	(66)
Barrows	Alfalfa hay	Free feeding	pH value at 45 min after slaughter ↑; pH value at 24 h after slaughter ↓; Monounsaturated fatty acids of longissimus lumborum muscle ↑; Saturated fatty acids in intramuscular fat of longissimus lumborum muscle ↓; Polyunsaturated fatty acids in the backfat ↑	(67)
Fattening pig	Alfalfa	50%	The percentage of weight gain, slaughter rate, backfat depth in the carcass ↓; The percentage of shoulder, ham and loin in the carcass ↑; The percentage of lean meat ↑	(68)
Growing-fattening pig	Alfalfa	50%	The proportion of lean meat in pork ↑	(69)
Fattening pigs	Alfalfa extract	0.2%	Growth and development ↑; Sensory evaluation scores for ham flavor and consistency ↑	(70)

## Possible mechanism of alfalfa improving pig's performance

Alfalfa is rich in nutrients, high-quality dietary fiber, protein with a balanced amino acid ratio, vitamins, mineral elements, and many other bioactive molecules. The beneficial effects of these nutrients on the health of pigs and pig production involve a variety of different pathways and mechanisms.

### Dietary fiber

#### Dietary fiber in alfalfa affects the physiology of the digestive tract

Dietary fiber is mainly composed of plant cell wall structural elements that are resistant to digestive enzymes and, therefore, not easily digested and decomposed in the upper digestive tract of animals (88). Furthermore, dietary fiber has a certain water-holding capacity (89). Recent studies have found that dietary fiber in alfalfa passes through the gastrointestinal tract and exerts several beneficial physiological effects (90). On the one hand, dietary fiber in alfalfa is not easily decomposed by digestive enzymes and can thus enhance gastrointestinal tract motility, promote gastrointestinal development, and stimulate digestive juice secretion (91). On the other hand, the water-holding capacity of dietary fiber can help reduce constipation (44).

#### Dietary fiber in alfalfa influences the intestinal microbiota of pigs

The alfalfa meal used as a fiber source in pig feed mainly undergoes fermentation by the microbiota in the large intestine of pigs. With further investigation, it is found that there is a close relationship between intestinal microbiota and intestinal barrier function and inflammation (92), and the changes of microbiota composition can affect host health (93). Intestinal microbiota metabolism can have a significant impact on the homeostasis of the intestinal mucosa of the host, the proliferation and differentiation of intestinal epithelial cells, and the function of the intestinal barrier (94). Varel et al. found that the addition of alfalfa meal to the diet of gilts mainly increased the relative level of the cellulolytic bacterium *B. succinogenes* in the intestine (38). Liu et al. added alfalfa meal to sow diets in the late gestation period and significantly increased the relative abundance of anti-inflammatory bacteria such as *Prevotellaceae_NK3B31_group* and *Lachnospiraceae_NK4A136_group* and significantly reduced pro-inflammatory bacteria such as *Terrisporobacter, Desulfovibrio* and *Helicobacter* (50). Another study showed that the addition of alfalfa meal to piglet diets significantly increased the Shannon index of flora in the jejunum, increased the diversity of intestinal flora, significantly increased the relative abundance of *Bacillus, Oceanobacillus, Lactococcus, Enterococcus*, and *Exiguobacterium*, and significantly decreased the relative abundance of *Mycoplasma* (55). Sun et al. supplemented alfalfa meal to piglet diets and significantly increased the relative abundances of cellulolytic and anti-inflammatory bacteria in the intestinal tract (56). Mu et al. found that the addition of alfalfa meal to piglet diets significantly reduced the relative level of *Bacteroidetes* in the cecum, significantly increased the relative level of *Clostridium cluster* XIVa in the colon, and significantly increased the level of butyrate and total SCFAs in the proximal colon of piglets (95). And *Clostridium cluster* XIVa belonging to *Firmicutes* contains many kinds of butyrate-producing bacteria (96). Zhang et al. added appropriate amount of alfalfa to suckling piglets' supplements to improve the composition of the piglets' intestinal flora, increased the abundance of *Coprococcus eutactus*, reduced the abundance of potential pathogen *Streptococcus suis*, increased the production of butyric acid in the intestine (60). *Streptococcus suis* is a pathogenic bacteria which can cause many physical diseases in pigs such as meningitis, septicemia and arthritis (97). According to various studies, it is obvious that the addition of alfalfa meal to pig diets can increase the relative abundance of beneficial bacteria and decrease the relative abundance of harmful bacteria in the intestinal tract. Furthermore, the beneficial bacteria can produce SCFAs and other beneficial molecules through fermentation and metabolism, which can have further beneficial effects on the intestinal tract and even on the pig health. In addition, dietary fiber can directly influence the host bile acid metabolic process through the intestinal flora, reducing bile acid metabolic disorders and improving animal health (98).

#### Dietary fiber in alfalfa fermented by intestinal microbiota produces SCFAs to improve pig health

Dietary fiber can be metabolized and fermented by the microbiota in the cecum and colon to produce SCFAs (99). SCFAs are the main fermentation product of intestinal bacteria and can serve as a source of energy for animals. Rerat et al. found that pigs fed diets supplemented with 6.5% alfalfa meal produced SCFAs that could provide ~30% energy for the body (100). In addition, SCFAs can function as signaling molecules, and thereby modulate the physiological functions of intestinal epithelial cells, including exerting anti-inflammatory and other effects (101). The physiological functions of SCFAs in the intestine are mainly related to acetate, propionate, and, in particular, butyrate (102), which has an extremely important role in maintaining mucosal barrier function and regulating immune function (103). Other SCFAs such as valerate and caproate are present in only very small amounts in the intestine (104, 105).

SCFAs can influence the barrier function of intestinal epithelial cells through a variety of regulatory pathways, of which there are two main distinct mechanisms including G protein-coupled receptors (GPCRs) pathway and histone deacetylase (HDAC) regulatory pathway. On the one hand, SCFAs can modulate the immune system by binding to GPCRs (GPR43, GPR109A) and Olfr78 receptors (106), while SCFAs also can promote the synthesis of mucin and enhance intestinal barrier function (107). SCFAs also promote the differentiation of regulatory T cells (Tregs) and the production of interleukin 10 through the upregulation of forkhead box protein 3 (FOXP3) *via* GPR43 (108). On the other hand, SCFA may act as an inhibitor of HDAC and stimulate monocytes and neutrophils by inducing HDAC inhibition, resulting in the inhibition of nuclear transcription factor-kappa B (NF-κB) and thus reducing the production of pro-inflammatory cytokines (109). In addition, SCFAs can activate the absent in melanoma 2 (AIM2) and Nod-like receptor pyrin domain 3 (NLRP3) inflammasomes, further affecting interleukin 18 production and enhancing intestinal epithelial barrier function (110). Secretory immunoglobulin A (sIgA) is a major component of the protective mechanism of the intestinal mucosa, it was found that butyric acid also improves sIgA level in the intestinal mucosa (111) and reduces the adhesion of harmful molecules to the intestinal wall. Thus, SCFAs can enhance intestinal barrier function and improve the health of pigs, thereby also enhancing their production performance.

### High-quality protein and amino acids in alfalfa can improve health condition of pigs

Proteins and amino acids are essential for many physiological functions and the synthesis of physiologically functional molecules in the body. An inadequate supply of amino acids may affect vital functions and lead to decreased performance, while an excess of amino acids may lead to increased nitrogen excretion and negative effects on the external environment (112). Alfalfa contains high levels of proteins with a relatively balanced amino acid profile, which makes it a good source of feed protein (9). Myer et al. found that the supplementation of alfalfa protein concentrate to the diets of growing-finishing pigs led to excellent performance, indicating that alfalfa protein concentrate could serve as a good protein supplement (113). Similarly, Pietrzak and Grela found that the addition of alfalfa protein concentrate to the diets of growing-finishing pigs improved red blood cell indices and reduce total cholesterol and low-density lipoprotein levels, thereby enhancing the hypolipidemic activity of the body (114). The high-quality protein in alfalfa can improve animal health, consequently also enhancing pig growth performance.

### Rich vitamins and mineral elements in alfalfa can improve pig health

Vitamins and mineral elements are essential for pig growth and development (115). Alfalfa is rich in a variety of vitamins such as vitamins B, C, E, and K (15). Vitamin E exerts a beneficial effect on the growth performance of pigs (116) while also helping to reduce lipid oxidation in pork and to improve pork quality (117). Fat-soluble vitamins are mainly involved in tissue growth and maintenance whereas B vitamins and vitamin C normally serve as co-factors for metabolic purposes (118). Alfalfa is rich in mineral elements, especially calcium, potassium, and magnesium (16). Calcium plays a crucial role in bone development while potassium is required for the maintenance of electrolyte balance and neuromuscular function; magnesium is a co-factor for many enzyme systems as well as a component of bone, and can also help boost immunity (17, 119). These vitamins and mineral elements, abundantly present in alfalfa, play essential roles in maintaining the health of pigs.

### Bioactive molecules in alfalfa exert positive effects on growth performance and antioxidant activity of pigs

The addition of alfalfa to the diets of pigs improves their health and enhances growth performance owing to the presence of dietary fiber and bioactive molecules in alfalfa, which help improve the physiological function of the animals. Alfalfa saponins can enhance bile acid secretion in pigs and increase cholesterol excretion for better body lipid metabolism (120). Cui et al. found that alfalfa saponins also increased the activity of antioxidant enzymes in intestinal cells, reduced malondialdehyde and lactate dehydrogenase release in H_2_O_2_-induced cells, and resisted cellular oxidative damage by restoring glutathione homeostasis (121). Alfalfa flavonoids, one of the bioactive molecules of alfalfa, can also have the effect of enhancing the antioxidant function of the body (122). Alfalfa flavonoids were found to reduce serum malondialdehyde levels and increase superoxide dismutase activity, thereby enhancing the total antioxidant capacity of the body (123). Alfalfa polysaccharides can regulate the immunity of animal. One research found that alfalfa polysaccharides can promote immunoglobulin M production by B cells through the Toll-like receptor 4 and improve the immunity level of the body (124). Furthermore, the addition of alfalfa polysaccharides to the diet has positive effects on growth performance and antioxidant activity of the animal (125). Zhang et al. supplemented alfalfa polysaccharides to weaned piglet diets, which enhanced piglet intestinal development, improved amylase and protease activities in the small intestine, and increased the relative intestinal levels of *Lactobacillus*, thus improving piglet growth performance and intestinal health (81).

In brief, alfalfa is rich in a number of elements that exert beneficial effects on the body through a variety of mechanisms. These elements can comprehensively enhance the production performance and improve the health of pigs.

## Summary and outlook

**Figure 1:**
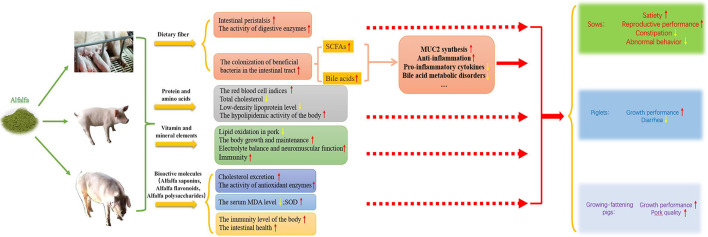
The effects and mechanisms of alfalfa on growth, reproductive performance, health condition and meat quality of pigs. Dietary fiber in alfalfa can stimulate intestinal peristalsis, increase the activity of digestive enzymes, and promote the colonization of beneficial bacteria in the intestinal tract, producing short-chain fatty acids through fermentation in the intestine and thus improving intestinal health; high quality protein and amino acids are beneficial to improve animal health condition; rich vitamins and mineral elements play an important role in various physiological functions of body; and bioactive molecules can improve the antioxidant and anti-inflammatory level. In this way, alfalfa supplementation in pig diets can improve reproductive performance of sows, growth performance of piglet and improve production performance and pork quality of growing-fattening pig.

As a high-quality feed source, alfalfa has been increasingly investigated and applied in pig production, and the optimum amount of alfalfa added to pig diets varies with the growth stage of the pig. The supplementation of dietary alfalfa to the diet of gestation sows can reduce constipation, alleviate abnormal behavior, improve satiety and reproductive performance; the supplementation of dietary alfalfa to the diet of piglets can improve growth performance and reduce diarrhea; the supplementation of dietary alfalfa to the diet of growing-fattening pigs can enhance production performance and pork quality (Figure 1). In addition, alfalfa contains many as yet unidentified bioactive molecules and growth factors and how these factors affect livestock and poultry still requires in-depth investigation, as do the underlying mechanisms. The determination of the appropriate amount of alfalfa to be added to the diets of pigs and how to best apply it in the pig farming industry will help to solve the current problems related to food competition between humans and livestock. The value of alfalfa as a feed supplement will continue to be explored to improve pig production as well as the quality and efficiency of pig husbandry.

## Author contributions

JM wrote the manuscript with input and guidance from YC and YS. WH, XY, JX, YZ, ZW, XZ, and CW helped to collect the literature. All authors contributed to the article and approved the submitted version.

## Funding

Financial support for this research was provided by Modern Agro-industry Technology Research System of China (CARS-34), Science and Technology Innovation Team of Henan Province High Quality Forage and Animal Health (No. 22IRTSTHN022), the Outstanding Talents of Henan Agricultural University (No. 30500636), Henan Provincial Science and Technology Research Project (No. 222102110007), China Postdoctoral Science Foundation (No. 2022M710046), and Henan Postdoctoral Science Foundation.

## Conflict of interest

The authors declare that the research was conducted in the absence of any commercial or financial relationships that could be construed as a potential conflict of interest.

## Publisher's note

All claims expressed in this article are solely those of the authors and do not necessarily represent those of their affiliated organizations, or those of the publisher, the editors and the reviewers. Any product that may be evaluated in this article, or claim that may be made by its manufacturer, is not guaranteed or endorsed by the publisher.

## References

[1] Koketsu Y Tani S Iida R. Factors for improving reproductive performance of sows and herd productivity in commercial breeding herds. Porc Heal Manag. (2017) 3:1–10. 10.1186/s40813-016-0049-7 28405457 PMC5382409

[2] Wang T Yao W Li J Shao Y He Q Xia J. Dietary garcinol supplementation improves diarrhea and intestinal barrier function associated with its modulation of gut microbiota in weaned piglets. J Anim Sci Biotechnol. (2020) 11:1–13. 10.1186/s40104-020-0426-6 32140225 PMC7050124

[3] Vermeulen L Van de Perre V Permentier L De Bie S Verbeke G Geers R. Pre-slaughter handling and pork quality. Meat Sci. (2015) 100:118–23. 10.1016/j.meatsci.2014.09.148 25460114

[4] Kulkarni KP Tayade R Asekova S Song JT Shannon JG Lee JD. Harnessing the potential of forage legumes, alfalfa, soybean, and cowpea for sustainable agriculture and global food security. Front Plant Sci. (2018) 9:1314. 10.3389/fpls.2018.01314 30283466 PMC6157451

[5] Radovic J Sokolovic D Markovic J. Alfalfa-most important perennial forage legume in animal husbandry. Biotechnol Anim Husb. (2009) 25:465–75. 10.2298/BAH0906465R

[6] Horner JL Bush LJ Adams GD Taliaferro CM. Comparative Nutritional Value of Eastern Gamagrass and Alfalfa Hay for Dairy Cows. J Dairy Sci. (1985) 68:2615–20. 10.3168/jds.S0022-0302(85)81145-0

[7] Vanzant ES Cochran RC. Performance and forage utilization by beef cattle receiving increasing amounts of alfalfa hay as a supplement to low-quality, tallgrass-prairie forage. J Anim Sci. (1994) 72:1059–67. 10.2527/1994.7241059x 8014141

[8] Marinova DH Ivanova II Zhekova ED. Evaluation of Romanian alfalfa varieties under the agro–environmental conditions in northern Bulgaria. Banat J Biotechnol. (2018) IX:56–64. 10.7904/2068-4738-IX(18)-56

[9] Blume L Hoischen-Taubner S Sundrum A. Alfalfa—a regional protein source for all farm animals. Landbauforschung. (2021) 71:1–13. 10.3220/LBF1615894157000

[10] Mattioli S Dal Bosco A Castellini C Falcinelli B Sileoni V Marconi O. Effect of heat- and freeze-drying treatments on phytochemical content and fatty acid profile of alfalfa and flax sprouts. J Sci Food Agric. (2019) 99:4029–35. 10.1002/jsfa.9630 30729526

[11] Boufaïed H Chouinard PY Tremblay GF Petit H V Michaud R Bélanger G. Fatty acids in forages. I factors affecting concentrations. Can J Anim Sci. (2003) 83:501–11. 10.4141/A02-098

[12] Whelan JFK. Linoleic Acid. Adv Nutr. (2013) 4:311–2. 10.3945/an.113.003772 23674797 PMC3650500

[13] Padovese R Curi R. Modulation of rat neutrophil function *in vitro* by cis- and trans-MUFA. Br J Nutr. (2009) 101:1351–9. 10.1017/S0007114508076307 18828952

[14] Grela ER Knaga S Winiarska-Mieczan A Zieba G. Effects of dietary alfalfa protein concentrate supplementation on performance, egg quality, and fatty acid composition of raw, freeze-dried, and hard-boiled eggs from Polbar laying hens. Poult Sci. (2020) 99:2256–65. 10.1016/j.psj.2019.11.030 32241511 PMC7587663

[15] Almuhayawi MS Hassan AHA Al Jaouni SK Alkhalifah DHM Hozzein WN Selim S. Influence of elevated CO2 on nutritive value and health-promoting prospective of three genotypes of Alfalfa sprouts (*Medicago Sativa*). Food Chem. (2021) 340:128147. 10.1016/j.foodchem.2020.128147 33032148

[16] Livia A Iorga S Mosouiu C Racovita RC Niculae OM Vlasceanu G. Rich source of nutrients for use in food. Int Sci Publ. (2017) 5:66–73.

[17] Gharibzahedi SMT Jafari SM. The importance of minerals in human nutrition: Bioavailability, food fortification, processing effects and nanoencapsulation. Trends Food Sci Technol. (2017) 62:119–32. 10.1016/j.tifs.2017.02.017

[18] Xie Y Wang L Sun H Wang Y Yang Z Zhang G. Immunomodulatory, antioxidant and intestinal morphology-regulating activities of alfalfa polysaccharides in mice. Int J Biol Macromol. (2019) 133:1107–14. 10.1016/j.ijbiomac.2019.04.144 31022488

[19] Jiang JF Song XM Huang X Wu JL Zhou WD Zheng HC. Effects of alfalfa meal on carcase quality and fat metabolism of Muscovy ducks. Br Poult Sci. (2012) 53:681–8. 10.1080/00071668.2012.731493 23281764

[20] Ouyang K Xu M Jiang Y Wang W. Effects of alfalfa flavonoids on broiler performance, meat quality, and gene expression. Can J Anim Sci. (2016) 96:332–41. 10.1139/cjas-2015-0132

[21] Hipsley EH. Dietary “fibre” and pregnancy toxaemia. Br Med J. (1953) 2:420–2. 10.1136/bmj.2.4833.420 13066743 PMC2029234

[22] Southgate DAT. Dietary fiber: analysis and food sources. Am J Clin Nutr. (1978) 31:S107–10. 10.1093/ajcn/31.10.S107 707357

[23] Dai FJ Chau CF. Classification and regulatory perspectives of dietary fiber. J Food Drug Anal. (2017) 25:37–42. 10.1016/j.jfda.2016.09.006 28911542 PMC9333437

[24] Meyer JH Lofgreen GP. Evaluation of alfalfa hay by chemical analyses. J Anim Sci. (1959) 18:1233–42. 10.2527/jas1959.1841233x

[25] Luo Y Liu Y Shen Y He J Li H Lan C. Fermented alfalfa meal instead of “grain-type” feedstuffs in the diet improves intestinal health related indexes in weaned pigs. Front Microbiol. (2021) 12:797875. 10.3389/fmicb.2021.797875 34966376 PMC8710769

[26] Jung HJG Lamb JAFS. Prediction of cell wall polysaccharide and lignin concentrations of alfalfa stems from detergent fiber analysis. Biomass Bioenergy. (2004) 27:365–73. 10.1016/j.biombioe.2004.04.001

[27] Robinson PH. Neutral detergent fiber (NDF) and its role in alfalfa analysis. 29th California Alfalfa Symposium. (1999). p. 60–7. Available online at: http://animalscience.ucdavis.edu/faculty/robinson/articles_folder/pdf/Web200001.PDF.

[28] Jung HG Engels FM. Alfalfa stem tissues: cell wall deposition, composition, and degradability. Crop Sci. (2002) 42:524–34. 10.2135/cropsci2002.5240

[29] Sharma BK Erdman RA Reeves JB. Rate and extent of in situ digestion of medium and high quality alfalfa and orchardgrass neutral detergent fiber as determined by extended periods of incubation time. J Dairy Sci. (1988) 71:3509–15. 10.3168/jds.S0022-0302(88)79958-0

[30] Jung HG Lamb JFS. Stem morphological and cell wall traits associated with divergent *in vitro* neutral detergent fiber digestibility in alfalfa clones. Crop Sci. (2006) 46:2054–61. 10.2135/cropsci2005.12.0470

[31] Jha R Fouhse JM Tiwari UP Li L Willing BP. Dietary fiber and intestinal health of monogastric animals. Front Vet Sci. (2019) 6:48. 10.3389/fvets.2019.00048 30886850 PMC6409295

[32] Hong J Ndou SP Adams S Scaria J Woyengo TA. Growth performance, visceral organ weights, and gut health of weaned pigs fed diets with different dietary fiber solubility and lipid sources. J Anim Sci. (2021) 99:skab292. 10.1093/jas/skab292 34657148 PMC8598925

[33] Wenk C. The role of dietary fibre in the digestive physiology of the pig. Anim Feed Sci Technol. (2001) 90:21–33. 10.1016/S0377-8401(01)00194-83040941

[34] Pu G Li P Du T Niu Q Fan L Wang H. Adding appropriate fiber in diet increases diversity and metabolic capacity of distal gut microbiota without altering fiber digestibility and growth rate of finishing pig. Front Microbiol. (2020) 11:533. 10.3389/fmicb.2020.00533 32328041 PMC7160236

[35] Nielsen TS Lærke HN Theil PK Sørensen JF Saarinen M Forssten S. Diets high in resistant starch and arabinoxylan modulate digestion processes and SCFA pool size in the large intestine and faecal microbial composition in pigs. Br J Nutr. (2014) 112:1837–49. 10.1017/S000711451400302X 25327182

[36] Le Goff G Noblet J Cherbut C. Intrinsic ability of the faecal microbial flora to ferment dietary fibre at different growth stages of pigs. Livest Prod Sci. (2003) 81:75–87. 10.1016/S0301-6226(02)00191-4

[37] Varel VH Yen JT. Microbial perspective on fiber utilization by swine. J Anim Sci. (1997) 75:2715–22. 10.2527/1997.75102715x 9331875

[38] Varel VH Robinson IM Jung HJG. Influence of dietary fiber on xylanolytic and cellulolytic bacteria of adult pigs. Appl Environ Microbiol. (1987) 53:22–6. 10.1128/aem.53.1.22-26.1987 3030194 PMC203595

[39] Mccallum IJ Ong S Mercer-Jones M. Chronic constipation in adults. BMJ. (2009) 338:b831. 10.1136/bmj.b831 19304766

[40] Neri I Blasi I Castro P Grandinetti G Ricchi A Facchinetti F. Polyethylene glycol electrolyte solution (Isocolan) for constipation during pregnancy: an observational open-label study. J Midwifery Women's Heal. (2004) 49:355–8. 10.1016/j.jmwh.2004.03.007 15236717

[41] Pearodwong P Muns R Tummaruk P. Prevalence of constipation and its influence on post-parturient disorders in tropical sows. Trop Anim Health Prod. (2016) 48:525–31. 10.1007/s11250-015-0984-3 26712363

[42] Oliviero C Heinonen M Valros A Peltoniemi O. Environmental and sow-related factors affecting the duration of farrowing. Anim Reprod Sci. (2010) 119:85–91. 10.1016/j.anireprosci.2009.12.009 20053511

[43] Baker DH Harmon BG Jensen AH. Value of alfalfa meal and wheat bran in diets for swine during prefarrowing and lactation. J Anim Sci. (1974) 39:838–40. 10.2527/jas1974.395838x 4473447

[44] Krogh U Bruun TS Amdi C Flummer C Poulsen J Theil PK. Colostrum production in sows fed different sources of fiber and fat during late gestation. Can J Anim Sci. (2015) 95:211–23. 10.4141/cjas-2014-060

[45] Björkman S Yun J Niku M Oliviero C Soede NM Peltoniemi OAT. Serial transvaginal ultrasound-guided biopsy of the porcine corpus luteum *in vivo*. Reprod Fertil Dev. (2017) 29:931–9. 10.1071/RD15435 28442044

[46] Holzgraefe DP Jensen AH Fahey GC Grummer RR. Effects of dietary alfalfa-orchardgrass hay and lasalocid on sow reproductive performance. J Anim Sci. (1986) 62:1145–53. 10.2527/jas1986.6251145x 3722007

[47] Libal GW Wahlstrom RC. Dehydrated Alfalfa in Sow and Gilt Gestation Diets. (1977).

[48] Calvert CC Steele NC Rosebrough RW. Digestibility of fiber components and reproductive performance of sows fed high levels of alfalfa meal. J Anim Sci. (1985) 61:595–602. 10.2527/jas1985.613595x 2999052

[49] Danielson DM Noonan JJ. Roughages in swine gestation diets. J Anim Sci. (1975) 41:94–9. 10.2527/jas1975.41194x

[50] Liu B Zhu X Cui Y Wang W Liu H Li Z. Consumption of dietary fiber from different sources during pregnancy alters sow gut microbiota and improves performance and reduces inflammation in sows and piglets. mSystems. (2021) 6:e00591–20. 10.1128/mSystems.00591-20 33500330 PMC7842364

[51] Teague HS. The influence of alfalfa on ovulation rate and other reproductive phenomena in gilts. J Anim Sci. (1955) 14:621–7. 10.1093/ansci/14.3.621

[52] Pollmann DS Danielson DM Crenshaw MA Peo ER. Long-term effects of dietary additions of alfalfa and tallow on sow reproductive performance. J Anim Sci. (1980) 51:294–9. 10.2527/jas1980.512294x

[53] Seerley RW Wahlstrom RC. Dehydrated alfalfa meal in rations for confined brood. SOWS. J Anim Sci. (1965) 24:448–53. 10.2527/jas1965.242448x 14324369

[54] Adams S Xiangjie K Hailong J Guixin Q Sossah FL Dongsheng C. Prebiotic effects of alfalfa (*Medicago sativa*) fiber on cecal bacterial composition, short-chain fatty acids, and diarrhea incidence in weaning piglets. RSC Adv. (2019) 9:13586–99. 10.1039/C9RA01251F 35519545 PMC9063875

[55] Liu B Wang W Zhu X Sun X Xiao J Li D. Response of gut microbiota to dietary fiber and metabolic interaction with SCFAs in piglets. Front Microbiol. (2018) 9:2344. 10.3389/fmicb.2018.02344 30323803 PMC6172335

[56] Sun X Cui Y Su Y Gao Z Diao X Li J. Dietary fiber ameliorates lipopolysaccharide-induced intestinal barrier function damage in piglets by modulation of intestinal microbiome. mSystems. (2021) 6:e01374–20. 10.1128/mSystems.01374-20 33824201 PMC8547013

[57] Freire JPB Guerreiro AJG Cunha LF Aumaitre A. Effect of dietary fibre source on total tract digestibility, caecum volatile fatty acids and digestive transit time in the weaned piglet. Anim Feed Sci Technol. (2000) 87:71–83. 10.1016/S0377-8401(00)00183-8

[58] Moore RJ Kornegay ET Grayson RL Lindemann MD. Growth, nutrient utilization and intestinal morphology of pigs fed high-fiber diets. J Anim Sci. (1988) 66:1570–9. 10.2527/jas1988.6661570x 2840428

[59] Stanley DL Ewan RC. Utilization of enery of hominy feed and alfalfa meal by young pigs. J Anim Sci. (1982) 54:1175–80. 10.2527/jas1982.5461175x

[60] Zhang L Mu C He X Su Y Mao S Zhang J Smidt H Zhu W. Effects of dietary fibre source on microbiota composition in the large intestine of suckling piglets. FEMS Microbiol Lett. (2016) 363:fnw138. 10.1093/femsle/fnw138 27231242

[61] Škrlep M Candek-Potokar M TomaŽin U Batorek Lukač N Flores M. Properties and aromatic profile of dry-fermented sausages produced from Krškopolje pigs reared under organic and conventional rearing regime. Animal. (2018) 12:1316–23. 10.1017/S1751731117002713 29070093

[62] Bohman VR Kidwell JF McCormick JA. High levels of alfalfa in the rations of growing-fattening swine. J Anim Sci. (1953) 12:876–80. 10.2527/jas1953.124876x

[63] Chen L Zhang HF Gao LX Zhao F Lu QP Sa RN. Effect of graded levels of fiber from alfalfa meal on intestinal nutrient and energy flow, and hindgut fermentation in growing pigs. J Anim Sci. (2013) 91:4757–64. 10.2527/jas.2013-6307 23965393

[64] Wang J Qin C He T Qiu K Sun W Zhang X. Alfalfa-containing diets alter luminal microbiota structure and short chain fatty acid sensing in the caecal mucosa of pigs. J Anim Sci Biotechnol. (2018) 9:1–9. 10.1186/s40104-017-0216-y 29372054 PMC5769528

[65] Kozera W Karpiesiuk K Bugnacka D Falkowski J Milewska W. Production performance of pigs reared in different systems and fed increased energy content diets with or without green alfalfa. S Afr J Anim Sci. (2016) 46:70–6. 10.4314/sajas.v46i1.9

[66] Stevenson JW Davey RJ Hiner RL. Some effects of dietary levels of protein and alfalfa meal and of antibiotic supplementation on growth, feed efficiency and carcass characteristics in swine. J Anim Sci. (1960) 19:887–97. 10.2527/jas1960.193887x

[67] TomaŽin U Batorek-Lukač N Škrlep M Prevolnik-Povše M Candek-Potokar M. Meat and fat quality of krškopolje pigs reared in conventional and organic production systems. Animal. (2019) 13:1103–10. 10.1017/S1751731118002409 30289382

[68] Bohman VR Hunter JE McCormick J. The effect of graded levels of alfalfa and aureomycin upon growing-fattening swine. J Anim Sci. (1955) 14:499–506. 10.2527/jas1955.142499x

[69] Kidwell JF Hunter JE. The utilization of a high level of alfalfa by growing-fattening swine. J Anim Sci. (1956) 15:1067–71. 10.2527/jas1956.1541067x

[70] Karwowska M Dolatowski ZJ Grela ER. Effect of dietary supplementation with extracted alfalfa meal on oxidation stability of cooked ham. Polish J Food Nutr Sci. (2007) 57:271–4.

[71] Farmer C. The Gestating and Lactating Sow. Wageningen: Wageningen Academic Publishers (2015). p. 1–452. 10.3920/978-90-8686-803-2

[72] Shang Q Liu S Liu H Mahfuz S Piao X. Impact of sugar beet pulp and wheat bran on serum biochemical profile, inflammatory responses and gut microbiota in sows during late gestation and lactation. J Anim Sci Biotechnol. (2021) 12:1–14. 10.1186/s40104-021-00573-3 33879267 PMC8059298

[73] Meunier-Salaün MC Edwards SA Robert S. Effect of dietary fibre on the behaviour and health of the restricted fed sow. Anim Feed Sci Technol. (2001) 90:53–69. 10.1016/S0377-8401(01)00196-1

[74] Quesnel H Meunier-Salaün MC Hamard A Guillemet R Etienne M Farmer C. Dietary fiber for pregnant sows: influence on sow physiology and performance during lactation. J Anim Sci. (2009) 87:532–43. 10.2527/jas.2008-1231 18849389

[75] Varel VH Pond WG. Enumeration and activity of cellulolytic bacteria from gestating swine fed various levels of dietary fiber. Appl Environ Microbiol. (1985) 49:858–62. 10.1128/aem.49.4.858-862.1985 2988439 PMC238458

[76] Varel VH. Activity of fiber-degrading microorganisms in the pig large intestine. J Anim Sci. (1987) 65:488–96. 10.2527/jas1987.652488x 3040656

[77] Li Y He J Zhang L Liu H Cao M Lin Y. Effects of dietary fiber supplementation in gestation diets on sow performance, physiology and milk composition for successive three parities. Anim Feed Sci Technol. (2021) 276:114945. 10.1016/j.anifeedsci.2021.114945

[78] Loisel F Farmer C Ramaekers P Quesnel H. Effects of high fiber intake during late pregnancy on sow physiology, colostrum production, and piglet performance. J Anim Sci. (2013) 91:5269–79. 10.2527/jas.2013-6526 23989876

[79] Reese D Prosch A Travnicek DA Eskridge KM Reese DE. Dietary fiber in sow gestation diets - an updated review dietary fiber in sow gestation diets—an updated review. Nebraska Swine Rep. (2008) 1:14–18.

[80] Adams S Kong X Che D Qin G Jiang H. Effects of dietary supplementation of Alfalfa (*Medicago Sattva*) fibre on the blood biochemistry, nitrogen metabolism, and intestinal morphometry in weaning piglets. Appl Ecol Environ Res. (2019) 17:2275–95. 10.15666/aeer/1702_22752295

[81] Zhang CY Gan LP Du MY Shang QH Xie YH Zhang GG. Effects of dietary supplementation of alfalfa polysaccharides on growth performance, small intestinal enzyme activities, morphology, and large intestinal selected microbiota of piglets. Livest Sci. (2019) 223:47–52. 10.1016/j.livsci.2019.01.027

[82] Shi YH Wang J Guo R Wang CZ Yan XB Xu B. Effects of alfalfa saponin extract on growth performance and some antioxidant indices of weaned piglets. Livest Sci. (2014) 167:257–62. 10.1016/j.livsci.2014.05.032

[83] Guevarra RB Lee JH Lee SH Seok MJ Kim DW Kang BN. Piglet gut microbial shifts early in life: causes and effects. J Anim Sci Biotechnol. (2019) 10:1–10. 10.1186/s40104-018-0308-3 30651985 PMC6330741

[84] Kass ML Van Soest PJ Pond WG Lewis B McDowell RE. Utilization of dietary fiber from alfalfa by growing swine. I apparent digestibility of diet components in specific segments of the gastrointestinal tract1. J Anim Sci. (1980) 50:175–91. 10.2527/jas1980.501175x

[85] Grela ER Semeniuk W Florek M. Effects of protein-xanthophyll (px) concentrate of alfalfa additive to crude protein-reduced diets on nitrogen excretion, growth performance and meat quality of pigs. J Cent Eur Agric. (2008) 9:669–76.

[86] Karwowska M Dolatowski ZJ Grela ER. Influence of dietary supplementation with extracted alfalfa meal on meat quality. Proceedings of 54th International Congress of Meat Science and Technology. (2008).

[87] Quander-Stoll N Früh B Bautze D Zollitsch W Leiber F Scheeder MRL. Sire-feed interactions for fattening performance and meat quality traits in growing-finishing pigs under a conventional and an organic feeding regimen. Meat Sci. (2021) 179:108555. 10.1016/j.meatsci.2021.108555 34023676

[88] Selvendran RR Stevens BJH Du Pont MS. Dietary fiber: chemistry, analysis, and properties. Adv Food Res. (1988) 31:117–209. 10.1016/S0065-2628(08)60167-6 2833079

[89] Yamazaki E Murakami K Kurita O. Easy preparation of dietary fiber with the high water-holding capacity from food sources. Plant Foods Hum Nutr. (2005) 60:17–23. 10.1007/s11130-005-2537-9 15898355

[90] Hamaker BR Tuncil YE. A perspective on the complexity of dietary fiber structures and their potential effect on the gut microbiota. J Mol Biol. (2014) 426:3838–50. 10.1016/j.jmb.2014.07.028 25088686

[91] Brambillasca S Zunino P Cajarville C. Addition of inulin, alfalfa and citrus pulp in diets for piglets: influence on nutritional and faecal parameters, intestinal organs, and colonic fermentation and bacterial populations. Livest Sci. (2015) 178:243–50. 10.1016/j.livsci.2015.06.003

[92] Yang J Qian K Wang C Wu Y. Roles of probiotic lactobacilli inclusion in helping piglets establish healthy intestinal inter-environment for pathogen defense. Probiotics Antimicrob Proteins. (2018) 10:243–50. 10.1007/s12602-017-9273-y 28361445

[93] Kabeerdoss J Sankaran V Pugazhendhi S Ramakrishna BS. Clostridium leptum group bacteria abundance and diversity in the fecal microbiota of patients with inflammatory bowel disease: a case-control study in India. BMC Gastroenterol. (2013) 13:1–8. 10.1186/1471-230X-13-20 23351032 PMC3565871

[94] Havenaar R. Intestinal health functions of colonic microbial metabolites: a review. Benef Microbes. (2011) 2:103–14. 10.3920/BM2011.0003 21840809

[95] Mu C Zhang L He X Smidt H Zhu W. Dietary fibres modulate the composition and activity of butyrate-producing bacteria in the large intestine of suckling piglets. Int J Gen Mol Microbiol. (2017) 110:687–96. 10.1007/s10482-017-0836-4 28161736

[96] Van Den Abbeele P Belzer C Goossens M Kleerebezem M De Vos WM Thas O. Butyrate-producing clostridium cluster XIVa species specifically colonize mucins in an *in vitro* gut model. ISME J. (2013) 7:949–61. 10.1038/ismej.2012.158 23235287 PMC3635240

[97] Su Y Yao W Perez-Gutierrez ON Smidt H Zhu WY. Changes in abundance of Lactobacillus spp. and Streptococcus suis in the stomach, jejunum and ileum of piglets after weaning. FEMS Microbiol Ecol. (2008) 66:546–55. 10.1111/j.1574-6941.2008.00529.x 18554303

[98] Singh J Metrani R Shivanagoudra SR Jayaprakasha GK Patil BS. Review on bile acids: effects of the gut microbiome, interactions with dietary fiber, and alterations in the bioaccessibility of bioactive compounds. J Agric Food Chem. (2019) 67:9124–38. 10.1021/acs.jafc.8b07306 30969768

[99] Koh A De Vadder F Kovatcheva-Datchary P Bäckhed F. From dietary fiber to host physiology: Short-chain fatty acids as key bacterial metabolites. Cell. (2016) 165:1332–45. 10.1016/j.cell.2016.05.041 27259147

[100] Rérat A Fiszlewicz M Giusi A Vaugelade P. Influence of meal frequency on postprandial variations in the production and absorption of volatile fatty acids in the digestive tract of conscious pigs. J Anim Sci. (1987) 64:448–56. 10.2527/jas1987.642448x 3558150

[101] Kamada N Seo SU Chen GY Núñez G. Role of the gut microbiota in immunity and inflammatory disease. Nat Rev Immunol. (2013) 13:321–35. 10.1038/nri3430 23618829

[102] Cummings JH Pomare EW Branch HWJ Naylor CPE MacFarlane GT. Short chain fatty acids in human large intestine, portal, hepatic and venous blood. Gut. (1987) 28:1221–7. 10.1136/gut.28.10.1221 3678950 PMC1433442

[103] Sugihara K Morhardt TL Kamada N. The role of dietary nutrients in inflammatory bowel disease. Front Immunol. (2019) 9:3183. 10.3389/fimmu.2018.03183 30697218 PMC6340967

[104] Ríos-Covián D Ruas-Madiedo P Margolles A Gueimonde M De. los Reyes-Gavilán CG, Salazar N. Intestinal short chain fatty acids and their link with diet and human health. Front Microbiol. (2016) 7:185. 10.3389/fmicb.2016.00185 26925050 PMC4756104

[105] Yoshikawa S Araoka R Kajihara Y Ito T Miyamoto H Kodama H. Valerate production by Megasphaera elsdenii isolated from pig feces. J Biosci Bioeng. (2018) 125:519–24. 10.1016/j.jbiosc.2017.12.016 29331526

[106] Han R Ma Y Xiao J You L Pedisić S Liao L. The possible mechanism of the protective effect of a sulfated polysaccharide from Gracilaria Lemaneiformis against colitis induced by dextran sulfate sodium in mice. Food Chem Toxicol. (2021) 149:112001. 10.1016/j.fct.2021.112001 33482260

[107] Gaudier E Jarry A Blottière HM De Coppet P Buisine MP Aubert JP. Butyrate specifically modulates MUC gene expression in intestinal epithelial goblet cells deprived of glucose. Am J Physiol Gastrointest Liver Physiol. (2004) 287:G1168–74. 10.1152/ajpgi.00219.2004 15308471

[108] Keshteli AH Madsen KL Dieleman LA. Diet in the pathogenesis and management of ulcerative colitis: a review of randomized controlled dietary interventions. Nutrients. (2019) 11:1498. 10.3390/nu11071498 31262022 PMC6683258

[109] Ratajczak W Rył A Mizerski A Walczakiewicz K Sipak O Laszczyńska M. Immunomodulatory potential of gut microbiome-derived shortchain fatty acids (SCFAs). Acta Biochim Pol. (2019) 66:1–12. 10.18388/abp.2018_2648 30831575

[110] Markowiak-Kopeć P Slizewska K. The effect of probiotics on the production of short-chain fatty acids by human intestinal microbiome. Nutrients. (2020) 12:1107. 10.3390/nu12041107 32316181 PMC7230973

[111] Murakoshi S Fukatsu K Omata J Moriya T Noguchi M Saitoh D. Effects of adding butyric acid to PN on gut-associated lymphoid tissue and mucosal immunoglobulin a levels. J Parenter Enter Nutr. (2011) 35:465–72. 10.1177/0148607110387610 21467244

[112] van Milgen J Dourmad JY. Concept and application of ideal protein for pigs. J Anim Sci Biotechnol. (2015) 6:1–11. 10.1186/s40104-015-0016-1 25937926 PMC4416387

[113] Myer RO Cheeke PR Kennick WH. Utilization of alfalfa protein concentrate by swine. J Anim Sci. (1975) 40:885–91. 10.2527/jas1975.405885x

[114] Pietrzak K Grela ER. Influence of alfalfa protein concentrate dietary supplementation on blood parameters of growing-finishing pigs. Bull Vet Inst Pulawy. (2015) 59:393–9. 10.1515/bvip-2015-0058

[115] Gaudré D Quiniou N. What mineral and vitamin levels to recommend in swine diets? Rev Bras Zootec. (2009) 38:190–200. 10.1590/S1516-35982009001300019

[116] Asghar A Gray JI Miller ER Ku P. -K, Booren AM, Buckley DJ. Influence of supranutritional vitamin E supplementation in the feed on swine growth performance and deposition in different tissues. J Sci Food Agric. (1991) 57:19–29. 10.1002/jsfa.2740570103

[117] Buckley DJ Morrissey PA Gray JI. Influence of dietary vitamin E on the oxidative stability and quality of pig meat. J Anim Sci. (1995) 73:3122–30. 10.2527/1995.73103122x 8617685

[118] Mahan DC Kim YY. The role of vitamins and minerals in the production of high quality pork—review. Asian Aust J Anim Sci. (1999) 12:287–94. 10.5713/ajas.1999.287

[119] Miller ER Kornegay ET. Mineral and vitamin nutrition of swine. J Anim Sci. (1983) 57 Suppl 2:315–29. 6352588

[120] Gaweł E Grzelak M. The effect of a protein-xanthophyll concentrate from alfalfa (phytobiotic) on animal production—a current review. Ann Anim Sci. (2012) 12:281–9. 10.2478/v10220-012-0023-5

[121] Cui Y Liu B Sun X Li Z Chen Y Guo Z. Protective effects of alfalfa saponins on oxidative stress-induced apoptotic cells. Food Funct. (2020) 11:8133–40. 10.1039/D0FO01797C 32869827

[122] Dabbou S Gasco L Rotolo L Pozzo L Tong JM Dong XF. Effects of dietary alfalfa flavonoids on the performance, meat quality and lipid oxidation of growing rabbits. Asian Aust J Anim Sci. (2018) 31:270–7. 10.5713/ajas.17.0284 28728357 PMC5767510

[123] Chen S Li X Liu X Wang N An Q Ye XM. Investigation of chemical composition, antioxidant activity, and the effects of alfalfa flavonoids on growth performance. Oxid Med Cell Longev. (2020) 2020:1–11. 10.1155/2020/8569237 32104541 PMC7035581

[124] Xie Y Wang L Sun H Shang Q Wang Y Zhang G. polysaccharide extracted from alfalfa activates splenic B cells by TLR4 and acts primarily: via the MAPK/p38 pathway. Food Funct. (2020) 11:9035–47. 10.1039/D0FO01711F 33021613

[125] Liu HW Dong XF Tong JM Zhang Q. Alfalfa polysaccharides improve the growth performance and antioxidant status of heat-stressed rabbits. Livest Sci. (2010) 131:88–93. 10.1016/j.livsci.2010.03.004

